# 46 Bardoxolone-methyl Microparticles Ameliorate Immune Dysfunction Following Burn and Inhalation Injury Through the NRF2/KEAP1 Axis

**DOI:** 10.1093/jbcr/irac012.049

**Published:** 2022-03-23

**Authors:** Roland F Seim, Rob Maile, Hannah R Hall, Shannon Wallet, Booker King

**Affiliations:** University of North Carolina at Chapel Hill, Chapel Hill, North Carolina; University of North Carolina at Chapel Hill, Chapel Hill, North Carolina; University of North Carolina at Chapel Hill, Chapel Hill, North Carolina; University of North Carolina at Chapel Hill, Chapel Hill, North Carolina; UNC Jaycee Burn Center, Chapel Hill, North Carolina

## Abstract

**Introduction:**

Severe burn injury leads to many systemic stresses that can seriously impact multiple systems throughout the body. These stresses are further exacerbated if there is a combined burn and inhalation injury, which leads to increased morbidity and mortality for many patients. Combined burn and inhalation injury causes an intense systemic inflammatory response and activation of the innate immune system which can lead to inflammatory complications, such as systemic inflammatory response syndrome and multiple organ failure. Nuclear Factor-Erythroid-2-Related Factor (NRF2) is a transcription factor that acts to downregulate overt damaging pro-inflammatory and oxidative responses and maintain immune homeostasis. This transcription factor remains bound to Kelch-like ECH-associated protein 1 (KEAP1) in the cytoplasm. Under oxidative stress, NRF2 dissociates from KEAP1 and translocates to the nucleus where it facilitates the transcription of anti-inflammatory and antioxidant genes. We hypothesized that NRF2 is a key regulator after burn and inhalation injury, and activation of NRF2 can limit the severity of burn and inhalation injury.

**Methods:**

To test this, we have developed a mouse model of combined cutaneous burn and woodsmoke inhalation injury. After burn and inhalation injury, we found that NRF2^-/-^ knockout mice have higher mortality compared to wild-type (WT) mice and suffer from increased vascular permeability and lung edema, suggesting that this transcription factor is important for controlling morbidity and mortality. In WT mice, NRF2 is activated following burn and inhalation injury, however, based on immunohistochemical staining, it is not sufficiently induced following this insult since it remains in the cytoplasm and is unable to transcribe anti-inflammatory genes. Therefore, we treated WT mice with an intraperitoneal injection of bardoxolone-methyl microparticles, a NRF2 activator that separates KEAP1 from NRF2 in the cytoplasm, immediately after burn and inhalation injury in WT mice

**Results:**

We observed significant decreases in mortality as well as reduced concentrations of certain pro-inflammatory cytokines in the blood and bronchoalveolar-lavage fluid of these mice. In the lungs of these mice, there was an upregulation in numerous pathways involved in the management of inflammation and immune response compared to mice that did not receive bardoxolone-methyl microparticles.

**Conclusions:**

In conclusion, treatment with bardoxolone-methyl microparticles immediately after burn and inhalation injury might be effective for reducing the severity of the inflammatory response and limiting inflammatory complications.

